# Intraspecific functional diversity of common species enhances community stability

**DOI:** 10.1002/ece3.2721

**Published:** 2017-02-08

**Authors:** Connor M. Wood, Shawn T. McKinney, Cynthia S. Loftin

**Affiliations:** ^1^ Department of Wildlife, Fisheries, and Conservation BiologyUniversity of MaineOronoMEUSA; ^2^ U.S. Geological SurveyMaine Cooperative Fish and Wildlife Research UnitOronoMEUSA; ^3^ Present address: Department of Forest and Wildlife EcologyUniversity of Wisconsin‐MadisonMadison WIUSA

**Keywords:** Community ecology, functional diversity, small mammals, stability, stable isotopes

## Abstract

Common species are fundamental to the structure and function of their communities and may enhance community stability through intraspecific functional diversity (iFD). We measured among‐habitat and within‐habitat iFD (i.e., among‐ and within‐plant community types) of two common small mammal species using stable isotopes and functional trait dendrograms, determined whether iFD was related to short‐term population stability and small mammal community stability, and tested whether spatially explicit trait filters helped explain observed patterns of iFD. Southern red‐backed voles (*Myodes gapperi*) had greater iFD than deer mice (*Peromyscus maniculatus*), both among habitats, and within the plant community in which they were most abundant (their “primary habitat”). *Peromyscus maniculatus* populations across habitats differed significantly between years and declined 78% in deciduous forests, their primary habitat, as did the overall deciduous forest small mammal community. *Myodes gapperi* populations were stable across habitats and within coniferous forest, their primary habitat, as was the coniferous forest small mammal community. Generalized linear models representing internal trait filters (e.g., competition), which increase within‐habitat type iFD, best explained variation in * M. gapperi* diet, while models representing internal filters and external filters (e.g., climate), which suppress within‐habitat iFD, best explained *P. maniculatus* diet. This supports the finding that *M. gapperi* had higher iFD than *P. maniculatus* and is consistent with the theory that internal trait filters are associated with higher iFD than external filters. Common species with high iFD can impart a stabilizing influence on their communities, information that can be important for conserving biodiversity under environmental change.

## 1 Introduction

Diversity begets ecological stability (McCann, 2000), and functional diversity plays a greater role in determining ecosystem processes, such as nitrogen fixation and control of agricultural pests, than species richness (McCann, 2000; Tilman et al., 1997). Nevertheless, species identity is important: Common species are fundamental to the structure and function of their communities, and even declines that do not result in extirpation can significantly affect ecosystem function, as the declines in bison (*Bison bison*) and cod (*Gadus morhua*) have reshaped the American Great Plains and North Atlantic Ocean, respectively (Gaston & Fuller, 2007). A single common species that contributes multiple functional groups to a community may stabilize its community in the face of changing environmental conditions (Bolnick et al., 2011). The intraspecific functional diversity, or individual‐level functional diversity (iFD; Cianciaruso, Batalha, Gaston, & Petchey, 2009) of common species, therefore, represents an important aspect of population and community dynamics. Do common species with high iFD stabilize their communities?

When environmental conditions change, most individuals in populations with low iFD will respond similarly, and population size may change suddenly and dramatically. Alternatively, when a population with greater iFD experiences the same change in environmental conditions, only some individuals will respond, and population fluctuations will be less pronounced (Bolnick et al., 2011; McCann, 2000; Scheffer et al., 2012). Common species play an outsized role in determining the dynamics of their communities, even if only by virtue of their sheer abundance. Thus, common species with high iFD could impart a stabilizing effect on community dynamics.

In the context of community stability, trait variation, rather than mean trait values, is of interest (Araújo, Bolnick, & Layman, 2011; Bolnick et al., 2011; Violle et al., 2012), and greater iFD corresponds with increased trait variation. For example, a population of a nonvolant small mammal species living in a given plant community may share greater trait similarity with a population living in the same plant community type, or habitat, on a geographically distant mountain than they will with a population living on the same mountain but in a different habitat. Thus, they may have high iFD (1) among habitats, but not within habitats; (2) within habitats, but not among habitats; or (3) both within and among habitats. Unless otherwise stated, we hereafter use “iFD” to refer to within‐habitat iFD, because it is the most closely associated with population stability.

Violle et al. (2012) introduced the concept of external and internal filters to make explorations of trait variation spatially explicit. This theoretical approach may also be useful for investigations into iFD, potentially providing a means to identify mechanisms contributing to patterns of iFD. External filters are processes that operate at a spatial extent larger than that of the target population or community and decrease local trait variation and thus iFD. For example, climatic conditions are generally an external filter that may select for a set of physiological traits for an entire population. Internal filters are processes that operate within a population or community and increase local trait variation and thus increase iFD. For example, competition for food is an internal filter that may increase dietary variation within a population. If external filters are the dominant process governing a given trait, species will have low trait variation within populations and thus low iFD. Alternatively, if internal filters are the dominant process determining trait value, species will have high within‐population trait variation and thus high iFD.

Stable isotope analysis provides a tool to quantify iFD based on an individuals’ diet (Araújo et al., 2011; Bearhop, Adams, Waldron, Fuller, & MacLeod, 2004). We treated diet itself as a functional trait, which assumes that species consuming different resources are playing different ecological roles. Under this approach, a carnivorous organism and an omnivorous one are considered to have different functional roles by virtue of their patterns of consumption. This may limit the scope of inference, as diet is not necessarily linked to all, or even many, of an individual's phenotypes, but it is simple, and its assumptions are unlikely to be violated. For example, functional differences in rats and mice in degraded and intact areas of tropical forest were indicated by differences in δ^15^N (Nakagawa, Hyodo, & Nakashizuka, 2007). Small mammals’ hair represents their diet on a monthly timescale (Priestley, 1966), making cross‐sectional diet samples relatively insensitive to short‐term (e.g., daily) dietary variation (Bolnick, Yang, Fordyce, Davis, & Svanbäck, 2002).

We collected hair samples from two common small mammal species found along elevation gradients spanning multiple plant communities in Maine and New Hampshire, USA, to answer four questions about the connection between dietary variation and population and small mammal community stability: (1) Does among‐ and within‐habitat iFD, measured by δ^15^N and δ^13^C, vary between our focal species the deer mouse, *Peromyscus maniculatus*, and the southern red‐backed vole, *Myodes gapperi*? If the two species have different levels of iFD; (2) is greater within‐habitat iFD associated with increased stability between years? (3) Does greater within‐habitat iFD of a common species result in increased stability in the overall small mammal community between years? Finally (4) do external and internal trait filters explain patterns of iFD?

We predicted that (1) the two species would display different levels of iFD based on their life‐history differences; (2) the species with greater iFD would exhibit greater population stability between years; (3) a small mammal community numerically dominated by a species with greater iFD would have greater stability between years than a small mammal community dominated by a species with lower iFD; and (4) traits of the species with greater iFD would be regulated more by internal filters than by external filters. The relationship between iFD and community function has been explored with simulations and some field data (Cianciaruso et al., 2009). Our study represents a novel evaluation of these theories with a sampling design for mammals that is both temporally intensive and spatially extensive. If the iFD of common species stabilizes both populations and communities with respect to environmental change, it represents a subtle but important reason to incorporate common species into conservation planning.

## 2 Materials and Methods

### 2.1 Study site and sampling

We conducted the study in the Appalachian Mountains of Maine and New Hampshire in the northeastern United States (44.7°N, −70.8°W) during June, July, and August of 2014 and 2015. We recognized three dominant plant communities in the study area: deciduous forest (primarily sugar maple (*Acer saccharum*) and beech (*Fagus grandifolia*); 0–600 m asl), coniferous forest (red spruce (*Picea rubens*) and balsam fir (*Abies balsamea*); 200–1,100 m asl), and alpine tundra (grasses and rock; 1,100–1,800 m asl). We established 10 transects following hiking trails that spanned the three plant communities. We randomly located a trapping grid at 10 sites along those transects within both deciduous and coniferous forest and at six sites in alpine tundra (*N* = 26 sites). Trap grids were operated sequentially; 13 sites were sampled in 2014 and 13 were sampled in 2015, six of which were previously sampled in 2014.

**Figure 1:**
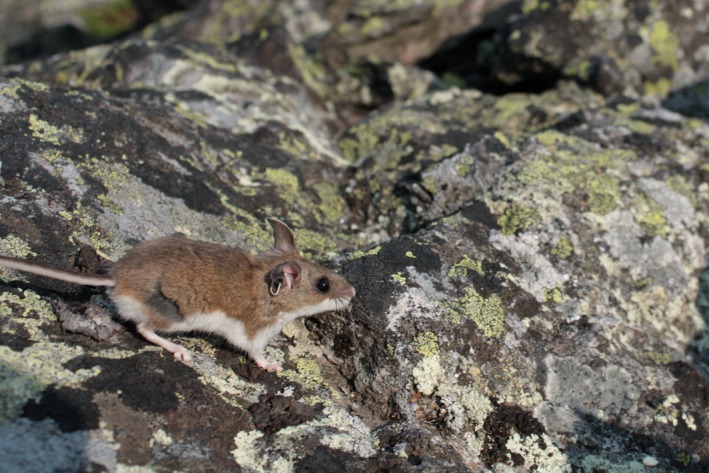
An adult deer mouse after an ear tag has been applied and a hair sample collected. Photograph by Connor Wood

At each trapping site, we placed 100 Sherman live traps (Sherman Trap Co., Tallahassee, FL) in a 90 m × 90 m grid (10 rows of 10 traps spaced 10 m apart), baited them with oats and peanuts, and supplied a cotton ball for nesting material. We checked traps in the morning (0700–1000) and evening (1630–1900) for three days and in the morning of a fourth day. We identified all individuals to species, applied a uniquely numbered ear tag (Kentucky Band and Tag Co., Newport, KY), measured head–body length and weight, clipped a lateral hair sample, and released them at the trap location (Figure 1).

Over both years, we collected 108, 35, and 25 samples from *P. maniculatus* in deciduous forest, coniferous forest, and alpine tundra, respectively (*N* = 166; two individuals from one site were excluded because their δ^15^N signatures were more than three *SD* beyond the mean). Over both years, we collected 42, 154, and three samples from *M. gapperi* in the three plant communities, respectively (*N* = 199). We analyzed hair samples for δ^15^N and δ^13^C signatures on a Finnigan Delta XP linked via a Conflow III to a Costech ECS 4010 Elemental Analyzer at the University of New Hampshire Stable Isotope Laboratory in Durham, New Hampshire, USA. Laboratory standards for isotopic measurements were NIST 1515, tuna, and a sporocarp (mushroom) standard. The average difference of duplicate samples was 0.35‰ for δ^15^N and 0.31‰ for δ^13^C.

### 2.2 Statistical analyses

We directly measured iFD with the total branch length of functional trait dendrograms (Cianciaruso et al., 2009; Petchey & Gaston, 2002, 2006). We first created a trait matrix with individuals in rows and functional traits, δ^15^N and δ^13^C, in columns for each species. We then converted the trait matrix into a distance matrix using Euclidean distance and clustered the distance matrix to create a dendrogram using an average linkage hierarchical clustering function. Finally, we computed the total branch length of the dendrogram, whereby longer branch length corresponds with greater iFD (Cianciaruso et al., 2009). We grouped the sites based on plant community type, which we refer to as “habitat,” and determined iFD both among and within habitats by conducting this analysis with 160 individuals randomly selected from across all habitats and 100 individuals randomly selected from within the habitat in which each species was most abundant (hereafter “primary habitat”; deciduous forest for *P. maniculatus* and coniferous forest for *M. gapperi*). By contrasting similar sympatric species rather than examining species individually, we mitigated the complexities of using stable isotopes to evaluate intraspecific diversity (Matthews & Mazumder, 2004).

We used an index, minimum number alive per 700 trap nights, to measure abundance at each site, and we compared abundance between years to evaluate short‐term population stability both among and within habitats. “Stability” entails several related traits, and given the limited duration of our study, we focused on constancy, the characteristic of remaining essentially unchanged (sensu Grimm & Wissel, 1997). For among‐habitat analyses, we pooled all trap sites within each habitat, and determined whether the relative abundance of each species across habitats changed between years with Chi‐square tests. We then tested for within‐habitat changes in each species abundance within their primary habitat with two‐sample, two‐tailed *t* tests. Finally, we defined small mammal “community size” as the sum of all individuals of all species captured in a given habitat type in a given year, and tested for an annual change in community size within deciduous forest and, separately, within coniferous forest with two‐sample, two‐tailed *t* tests.

We created generalized linear models of both stable isotope signatures for both species to determine whether external or internal filters were dominant. We concluded that external filters were dominant if among‐habitat models, which partitioned variation in stable isotope signatures by habitat, ranked highest in model selection. We treated habitat (i.e., plant community type) as a categorical, nominal variable. Alternatively, we concluded that internal filters were dominant if within‐habitat models, which partitioned variation in stable isotope signatures by individual trapping site, ranked highest in model selection. We treated trapping site as a categorical, nominal variable. We compared models with the Akaike information criterion with a correction for small sample size (AIC*c*) and ranked them by subtracting the lowest AIC*c* score from all others (*d*AIC*c*). Models with a *d*AIC*c* between 0 and 2 were considered to have substantial and comparable support from the data (Burnham & Anderson, 2010). For the among‐habitat models, we included habitat alone and with all first‐order combinations of the following: (1) the other stable isotope signature (i.e., δ^15^N when δ^13^C was the dependent variable and vice versa); (2) body condition (body‐mass residual; Schulte‐Hostedde, Zinner, Millar, & Hickling, 2005), and two temporal variables; (3) year, to account for changes in food resources between years; and (4) Julian date, to account for seasonal changes in resources. For within‐habitat models, we included trapping site alone and with all first‐order combinations of opposite stable isotope signature and body condition (as described in 1 and 2 above); year and date were implicit in trapping site. We did not incorporate the stable isotope signatures of food items, which precluded some methods of calculating individual specialization (Araújo, Bolnick, Machado, Giaretta, & dos Reis, 2007; Bolnick et al., 2002), because other studies of regional food resources had limited success with mixing models (Seger, Servello, Cross, & Keisler, 2013). Probabilistic statistical tests were evaluated at a 5% significance level. Analyses were performed with R version 3.0.3 (R Core Team 2014) and the vegan (Oksanen et al., 2013) and MuMIn (Bartoń, 2014) packages.

## 3 Results

**Figure 2:**
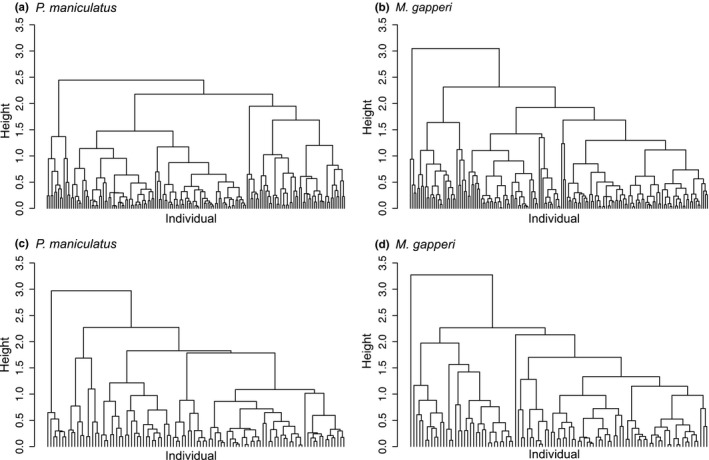
Dendrograms measuring the intraspecific functional diversity (iFD) of two species, *Peromyscus maniculatus* and *Myodes gapperi*, based on two traits, δ^15^N and δ^13^C, at two ecological scales, where greater total branch length corresponds with greater iFD (Cianciaruso et al., 2009; Petchey & Gaston, 2002, 2006). Among‐habitat analyses (a, b) compared individuals among all three plant community types: *P. maniculatus* branch length was 60.31, and *M. gapperi* branch length was 64.91 (*N* = 160 randomly selected individuals of each species). Within‐habitat analyses (c, d) compared individuals within the plant community in which each species was numerically dominant: *P. maniculatus* (deciduous forest) branch length was 45.43, and *M. gapperi* (coniferous forest) branch length was 51.32 (*N* = 100 randomly selected individuals of each species). Dendrograms were based on trait matrices, which were converted to Euclidean distance matrices, and then clustered with an average linkage hierarchical clustering function

Visual inspection of normal QQ plots of δ^15^N and δ^13^C for both species indicated no substantial deviations from normality. Total branch length of a functional dendrogram is a metric of functional diversity (Cianciaruso et al., 2009; Petchey & Gaston, 2002, 2006). The dendrogram of *M. gapperi* functional traits among habitats had a greater total branch length (64.91) than that of *P. maniculatus* (60.31). Similarly, the species’ primary habitat dendrograms indicated that *M. gapperi* functional traits had a greater total branch length (51.32) than that of *P. maniculatus* (45.34). Thus, *M. gapperi* had greater iFD than *P. maniculatus* both among and within habitats along the elevational gradient we surveyed (Figure 2). Although the within‐habitat analyses incorporated individuals occupying the same habitat type but from locations up to 120 km apart, preliminary analyses confirmed that the differences among sites within the same habitat type were far smaller than the differences between sites less than 10 km apart but located in different habitats.

*Peromyscus maniculatus* relative abundance across plant communities differed significantly between years (χ^2^ = 12.66, *df* = 2, *p* < .002). Its abundance within deciduous forest, its primary habitat, dropped 77.5% between years, and there was high variability in abundance among sites (t_5_ = 2.17, *p* = .08; Table 1). There was no change in *M. gapperi* relative abundance across plant communities (χ^2^ = 0.10, *df* = 2, *p* > .05), and its abundance in coniferous forest, its primary habitat, did not change significantly between years (t_5_ = −0.203, *p* > .05; Table 1), consistent with the prediction that the species with greater iFD would be more stable between years.

**Table 1:** Total changes in minimum number alive per unit effort between years, with percent change listed parenthetically, of small mammals sampled at 20 sites in Maine and New Hampshire, USA, during June–August 2014 and 2015. The small mammal community in deciduous forest^a^ changed significantly between years (t_5_ = 2.89, *p* < .038), whereas the small mammal community in coniferous forest did not change between years (t_5_ = 0.543, *p* > .05)

	2014		2015	
	Deciduous	Coniferous	Deciduous	Coniferous
*Peromyscus maniculatus*	102	22	23 (−77.5)	18 (−18.2)
*Myodes gapperi*	25	96	28 (12.0)	104 (8.3)
*Napaeozapus insignis*	118	27	33 (−72.0)	6 (−77.8)
*Blarina brevicauda*	21	8	0 (−100.0)	1 (−87.5)
*Tamius striatus*	14	7	0 (−100.0)	0 (−100.0)
*P. leucopus*	9	2	0 (−100.0)	0 (−100.0)
*Sorex cinereus*	1	0	0 (−100.0)	0 (0.0)
*M. pennsylvanicus*	1	2	1 (0.0)	3 (50.0)
Total	173^a^	164	52^a^ (−69.9)	132 (−20.0)

a *N. insignis* abundance was excluded from the deciduous forest community totals to isolate the effect of *P. maniculatus* population fluctuations on community change.

The small mammal communities in both deciduous forest and coniferous forest were composed of eight species in varying proportions: *P. maniculatus*,* M. gapperi*, woodland jumping mice (*Napaeozapus insignis*), short‐tailed shrews (*Blarina brevicauda*), eastern chipmunks (*Tamius striatus*), white‐footed mice (*P. leucopus*), masked shrews (*Sorex cinereus*), and meadow voles (*M. pennsylvanicus*). On average, *P. maniculatus* and *N. insignis* each accounted for 21%–39% of the deciduous forest small mammal community size, and both experienced steep declines between the two sample years (−77.5% and −72.0%, respectively; Table 1). We isolated the effect of *P. maniculatus* population fluctuations on small mammal community variability by subtracting *N. insignis* abundance from the deciduous forest small mammal community abundance. After that correction, *P. maniculatus* comprised 38%–58% of the individuals, and was the only such abundant species in deciduous forest. This modified deciduous forest small mammal community declined significantly between years (t_5_ = 2.89, *p* = .037; −69.0% change; Table 1). *Myodes gapperi* accounted for 51%–100% of coniferous forest small mammal community. That community showed a smaller and nonsignificant decline between years (−20.0%; t_5_ = −0.203, *p* > .05; Table 1). *M. gapperi* and *M. pennsylvanicus* increased between years, although only *M. gapperi* was abundant enough to meaningfully ameliorate the declines of the other six species (Table 1). Consistent with our prediction and prevailing theory (Gaston & Fuller, 2007), changes in the small mammal community reflected changes in the population of its most abundant species: The deciduous forest community declined significantly when the *P. maniculatus* population declined, whereas the coniferous forest small mammal community did not change significantly due to the constancy of the *M. gapperi* population.

Model selection indicated support for both internal and external filters regulating *P. maniculatus* diet. Within‐habitat models, which represented internal filters, performed best for δ^15^N; two top models of δ^13^C represented the among‐habitat, or external filter, hypothesis, while the third represented within‐habitat variation (Table 2a). Superior performance of within‐habitat models suggests that internal filters dominated *M. gapperi* diet (Table 2b). The top‐ranked model for both stable isotope signatures included trapping site and body condition, while the second‐ranked model included those terms and the other stable isotope signature. The importance of internal filters in regulating *M. gapperi* diet in comparison with the combination of internal and external filters regulating *P. maniculatus* diet is consistent with *M. gapperi*'s moderately greater iFD.

**Table 2:** Top models (*d*AIC*c* < 7) for *Peromyscus maniculatus* (a) and *Myodes gapperi* (b) stable isotope signatures sampled in Maine and New Hampshire, USA (2014 and 2015) and sorted by AIC*c*. External filters are landscape‐scale processes that decrease local (within‐habitat) variation and were represented by a categorical, nominal variable for habitat (*N* = 3 plant community types; beta values not shown). Internal filters are local‐scale processes that increase within‐habitat variation and were represented by a categorical, nominal variable for trapping site (*N* = 26 locations; beta values not shown). Explanatory variables were *z*‐standardized. Condition is body‐mass residual

		Filter	Beta (*SE*)	AIC*c*	*d*AIC*c*	*w*
(a)	δ^15^N	Internal	Condition × 0.12 (0.057)	383.8	0	0.80
		Internal	Condition × 0.12(0.057) + δ^13^C × 0.007(0.065)	386.6	2.8	0.20
	δ^13^C	External	– Year × 0.56(0.12) + δ^15^N*0.11(0.048) − Condition × 0.035 (0.005)	290.7	0	0.37
		Internal	– Condition × 0.049 (0.051)	291.6	0.9	0.24
		External	– Year*0.59(0.13) + δ^15^N × 0.096(0.056) − Condition × 0.041(0.051) – Day × 0.0024(0.0030)	292.2	1.6	0.17
(b)	δ^15^N	Internal	Condition *0.042(0.059)	568.5	0	0.54
		Internal	– δ^13^C*0.10(0.070) + Condition × 0.050(0.059)	568.8	0.3	0.46
	δ^13^C	Internal	Condition × 0.84 (0.067)	288.0	0	0.54
		Internal	– δ^15^N × 0.13(0.089) + Condition × 0.090(0.067)	288.3	0.3	0.46

## 4 Discussion

Species responding to internal or local filters (e.g., competition) are expected to have elevated iFD, which should lead to increased population stability (McCann, 2000; Tilman et al., 1997) relative to sympatric species responding to external filters (e.g., climate) (Violle et al., 2012). Our results generally supported these predictions. There were differences in iFD between the two focal species, with *M. gapperi* exhibiting higher iFD than *P. maniculatus* both among habitats and within the species’ respective primary habitats. Furthermore, *M. gapperi* populations were stable between years as was the small mammal community in their primary habitat (coniferous forest), while populations of *P. maniculatus*, which had lower iFD, declined dramatically between years. Consequently, the overall small mammal community in deciduous forest, the primary habitat of *P. maniculatus*, also declined. Differences in iFD may be driven by the species’ responses to trait filters. Internal filters regulated *M. gapperi* diet, whereas a combination of external and internal filters regulated *P. maniculatus* diet. Our hypothesis that common species with greater iFD may buffer their communities against environmental change that affects abundance is supported by our results.

The potential for sudden, drastic state shifts increases as more components in a complex system share stressors (Scheffer et al., 2012). *Myodes gapperi* displayed greater iFD than *P. maniculatus*: the dietary diversity of *M. gapperi* reduces the effects of fluctuations in single food items, whereas the diet of *P. maniculatus* was relatively homogeneous and thus more sensitive to changes in fewer food resources. The mast event of a single tree species can elicit a large numerical response in *P. maniculatus* populations (Jensen, Demers, McNulty, Jakubas, & Humphries, 2012), whereas a comparable fluctuation in *M. gapperi* populations would require a simultaneous decline in several food resources. This link between dietary variation and stability has been demonstrated in other systems. Stellar sea lion (*Eumetopias jubatus*) populations in Alaska with simple diets experienced greater declines than those with greater dietary diversity (Merrick, Chumbley, & Byrd, 1997). Likewise, tropical bird species in Borneo with wide dietary niches were more likely to persist after disturbance than those with narrow or inflexible feeding habits (Edwards et al., 2013). Including dietary variation and uncertainty, as our approach does, improves models of ecosystem functioning (Grêt‐Regamey, Brunner, Altwegg, & Bebi, 2013).

The relative importance of external filters on *P. maniculatus* populations and internal filters on *M. gapperi* populations are consistent with their regional population dynamics. *Peromyscus maniculatus* populations cycle in response to mast events (Jensen et al., 2012), which create pulses of food in such abundance that competition is reduced. Two of the three top models of *P. maniculatus* δ^13^C included habitat and year, and one also included a date term, which is consistent with cyclically fluctuating resources such as mast events in deciduous forest, an external filter, influencing their diet. Although *M. gapperi* also responds to mast events (Jensen et al., 2012), their regional populations, and vole populations globally, display complex population cycling that is driven in part by intraspecific density‐dependent factors, which are internal filters (Lima, Berryman, & Stenseth, 2006; Merritt, Lima, & Bozinovic, 2001). The stability of small mammal populations is important because small mammals exert top‐down pressure on plant communities (Bricker, Pearson, & Maron, 2010) and bottom‐up pressure on mesocarnivore populations (Jensen et al., 2012), and they potentially mediate interactions between mesocarnivores and forest structure (Fuller & Harrison, 2005). This underscores the value of understanding the stability of populations of common species for conserving biodiversity.

Our results are not without some ambiguity. The differences in iFD between the two species were not extreme, which is not surprising given that both species reflected the influence of internal filters. Increased differentiation in iFD would be expected if one species were regulated entirely by external filters and the other by internal filters. Greater insight into the species’ functional roles could be gained by sampling additional stable isotope sources because fractionation rates differ among tissue types (Tieszen, Boutton, Tesdahl, & Slade, 1983). Additionally, considering more functional traits may enhance our understanding of iFD (Cianciaruso et al., 2009). Furthermore, it is difficult to separate species effects from habitat effects, as the two species occupied different primary habitats. It is unlikely that the differences we observed are due to habitat alone, because *P. maniculatus* declined in both deciduous and coniferous forest between 2014 and 2015, while *M. gapperi* increased in both habitats over the same period (Table 1). For the same reason, changes in predator density are unlikely to have driven the patterns of abundance we observed. We did not collect hair samples from *N. insignis* because previous research suggested that they were rare in that area (Fuller, Harrison, & Lachowski, 2004). Incorporating them into subsequent studies would be a valuable step toward addressing some of these issues. More broadly, controlling for habitat and experimentally manipulating the abundance of common species is a logical next step to this line of research (e.g., Brunner et al., 2013), albeit a resource‐intensive one that was beyond the scope of our study.

Incorporating functional diversity is essential to understanding community dynamics (Hulot, Lacroix, Lescher‐Moutoué, & Loreau, 2000), and stable isotopes have been used to measure iFD in terrestrial small mammals (Nakagawa et al., 2007), marine mammals (Yurkowski et al., 2015), tropical birds (Edwards et al., 2013), terrestrial invertebrates (Blüthgen, Gebauer, & Fiedler, 2003), and to map the trophic structure of entire communities (Layman, Arrington, Montaña, & Post, 2007). We used stable isotopes to quantify the functional diversity of abundant small mammal species, information that, in turn, accurately predicted population and community change between years, illustrating their utility as a tool for biodiversity conservation. Finally, the internal/external trait filter framework provided insight into underlying drivers of those patterns. Cianciaruso et al. (2009) suggested that incorporating iFD would enhance our understanding of the mechanisms that link individuals to ecosystem processes; our results represent a clear step in that direction.

## Data Accessibility

Capture totals and stable isotope values: uploaded as online supporting information.

## Conflict of Interest

None declared.
